# Formation and fluorescent mechanism of red emissive carbon dots from *o*-phenylenediamine and catechol system

**DOI:** 10.1038/s41377-022-00984-5

**Published:** 2022-10-13

**Authors:** Pengfei Li, Shanshan Xue, Lu Sun, Xupeng Zong, Li An, Dan Qu, Xiayan Wang, Zaicheng Sun

**Affiliations:** Center of Excellence for Environmental Safety and Biological Effects, Beijing Key Laboratory for Green Catalysis and Separation, Department of Chemistry, Beijing University of Technology, 100124 Beijing, China

**Keywords:** Nanoparticles, Optical properties and devices

## Abstract

Carbon dots (CDs) as the advancing fluorescent carbon nanomaterial have superior potential and prospective. However, the ambiguous photoluminescence (PL) mechanism and intricate structure-function relationship become the greatest hindrances in the development and applications of CDs. Herein, red emissive CDs were synthesized in high yield from *o*-phenylenediamine (oPD) and catechol (CAT). The PL mechanism of the CDs is considered as the molecular state fluorophores because 5,14-dihydroquinoxalino[2,3-b] phenazine (DHQP) is separated and exhibits the same PL properties and behavior as the CDs. These include the peak position and shape of the PL emission and PL excitation and the emission dependence on pH and solvent polarity. Both of them display close PL lifetime decays. Based on these, we deduce that DHQP is the fluorophore of the red emissive CDs and the PL mechanism of CDs is similar to DHQP. During the PL emission of CDs, the electron of the molecule state can transfer to CDs. The formation process of DHQP is further confirmed by the reaction intermediates (phthalazine, dimers) and oPD. These findings provide insights into the PL mechanism of this type of CDs and may guide the further development of tunable CDs for tailored properties.

## Abstract (web summary)

The fluorophore source of red emissive oPD&CAT system CDs is the 5,14-dihydroquinoxalino[2,3-b] phenazine (DHQP), formed in the initial reaction stage. Subsequently, the DHQP was connected with carbon core. The excited electrons of the molecular state can transfer into the energy level of CDs, which leads to UV excitation red emissive intensity decreased.

## Graphical abstract

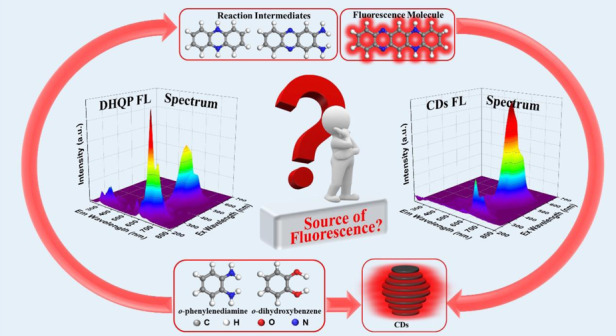

## Introduction

Carbon dots (CDs) as a new class of fluorescent carbon nanomaterial has attracted much more attention progress due to their low cost, high stability, low toxicity, high biocompatibility, easy surface modification, and tunable fluorescent properties since it was discovered in 2004^1–3^. Those unique properties enable it to have fantastic application potential in bioimaging, biological probe, biomedicine, catalysis, light-emitting diodes (LEDs), and so on^4–12^. Knowledge of their PL mechanisms is of significance in guiding the synthesis and promoting applications of CDs with tunable PL emissions. However, the intrinsic PL emission mechanism remains unclear, and a unified mechanism has not been achieved because of differences in particle structures. Thus, the revealing of CDs structure and PL mechanism is very significant in promoting its development and applications. Currently, the most acceptable PL mechanism can be described into three categories: surface state, carbon core state, and molecular state^2^. The carbon core state refers to the π–π* electron transitions of the conjugated sp^2^ domains^4,13,14^. The size of the isolated sp^2^ subdomain determines the emission of CDs^15^. The characteristics of the surface state are that the surface functional groups connecting with the carbon backbone control the electron structures and energy level by hybridization. The luminescence can be adjusted by altering types and contents of surface functional groups^16,17^ or the heteroatoms doping^18,19^. The molecule state refers to the molecular fluorophores or their aggregate which is connected with CDs dominated by the fluorescence, and it generally endows CDs with a strong PL and a high PLQY^20,21^. The CDs from citric acid (CA) are well investigated and analyzed, ICPA (imidazo[1,2-a]pyridine-7-carboxylic acid) was considered as a fluorescent molecule crosslinked with the CDs core, that is the strong blue PL center of the CDs from CA and ethylenediamine^20,22^. In general, different CDs synthesized by different routes possess structural features, they demonstrate different PL properties and mechanisms. However, the CDs obtained from aromatic molecules are rarely investigated.

Red emissive CDs not only can penetrate tissue deeply to avoid auto-fluorescence of organisms^5,23^, but also is a significant component of the white light-emitting diodes (WLEDs)^24^, it has been recognized as a key requirement to promote the practical applications of CDs in most fields^25^. Small aromatic molecules, as the common precursors, can generate large sp^2^ domains with a narrow energy gap, which is beneficial to tuning the emission and excitation toward long wavelength^26^. However, the PL mechanism of red emissive CDs prepared by aromatic molecules has always been controversial. Lin and coworkers through the solvothermal method synthesized red, green, and blue luminescence CDs with *p*-phenylenediamine (pPD), *o*-phenylenediamine (oPD), and *m*-phenylenediamine (mPD) as precursors, respectively. Simultaneously, the red emission for the CDs is attributed to the bigger particle size and more nitrogen content^27^. Xiong group used oPD as a precursor to prepare full-color emissive CDs by solvent-free approach, with the increased conclusion that the size of sp^2^-conjugated domains and the content of graphitic nitrogen contribute to red emission of CDs^28^. Zhong and coworkers supposed that newly emerged 2,3-diaminophenazine (DAPN) fluorophore interaction with carbon structure and its protonation directly determine the molecular state fluorescence of CDs^29^. Neeraj Soni et al. obtained red, green, and blue emissive components isolated and purified from CDs solution by column chromatographic. The red PL is attributed to a molecular fluorophore, quinoxalino[2,3-b]phenazine-2,3-diamine (QXPDA), the QXPDA attached to the surface of core carbon, and aggregated together, which led to green and blue emission^30^. Overall, the red PL emission mechanism, which keeps mystery, strongly depends on the chemical structure of fluorophores. The defined chemical structure will promote the understanding of the PL of CDs.

Herein, we synthesized red emissive CDs with high yield from oPD and catechol (CAT) catalyzed by aluminum chloride hexahydrates (AlCl_3_·6H_2_O) through the solvent-free method. To reveal the PL mechanism of red emissive CDs, the reaction intermediates are investigated by high-performance liquid chromatography-mass spectroscopy (HPLC-MS), proton nuclear magnetic resonance (^1^H NMR), and PL spectroscopies. It discloses that molecular organic species of 5,14-dihydroquinoxalino[2,3-b] phenazine (DHQP) is an important intermediate, which exhibits similar PL properties as CDs. That implies that DHQP is the fluorophore of red emissive CDs, the PL mechanism was proposed to follow the molecular state PL. Furthermore, the forming process of DHQP is proposed. The oPD and CAT form phthalazine and 2,3-diaminophenazine (2,3-DAPN) by dehydrated reaction, and then 2,3-DAPN and phenazine reaction with oPD to produce fluorescent molecule (DHQP). With prolonging the reaction time, graphitic CDs fragments gradually form by carbonizing these conjugated molecules, DHQP unit is remained during the carbonization, either incorporated into the carbon framework or linked to the surface of CDs. The remained DHQP unit on the carbon core, as the molecular fluorophore, emits a similar red light to the DHQP molecule. However, CDs exhibit higher solubility and photo and thermal stability than DHQP. In the fluorescence mechanism of CDs, the excited electron of DHQP can transfer to the CDs’ energy level, leading to the maximum excitation wavelength of CDs redshift to 540 nm. This discovery of the PL mechanism has specific directive significance for the synthesis and applications of CDs.

## Results

### Characterization of red emission CDs

**Fig. 1:**
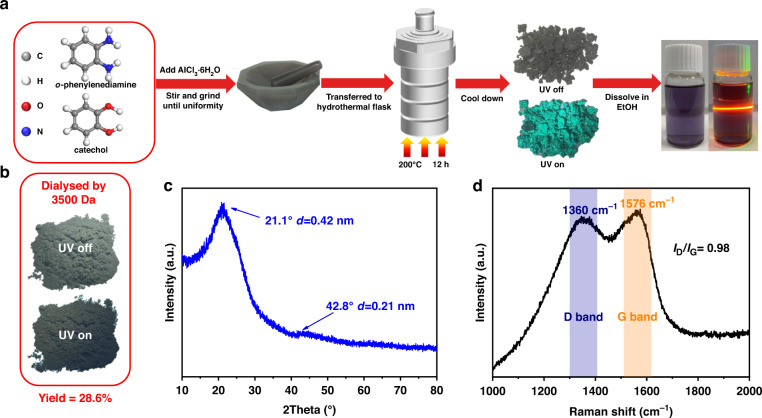
Synthesis of the red emissive CDs. **a** Schematics of the solvent-free synthesis of the red emissive CDs using oPD and CAT as precursor molecules. **b** Photo of the red emission CDs powder. **c** X-ray diffraction (XRD) pattern of the CDs powder. **d** Raman spectra of the CDs powder

In this work, red emissive CDs were synthesized from oPD and CAT as the precursors catalyzed by AlCl_3_ by the solvent-free method. As shown in Fig. 1a, the as-prepared product shows intense cyan solid-state fluorescence under ultraviolet light. However, the X-ray diffraction (XRD) pattern exhibits multiple sharp peaks (Fig. S1a), which indicates the as-prepared product contains a lot of crystalline species. The PL spectra exhibit two strong emissions at 600 nm and 650 nm under the excitation at 520 nm (Fig. S1b, c). To remove impurities, the as-prepared product was dissolved in ethanol for dialysis purification with 3500 Da molecular weight cut-off in DI water till no fluorescence was detected in dialysate. A gray powder can be obtained and lost its cyan solid-state fluorescence (Fig. 1b) after dialysis. The yield of CDs prepared by this method can reach 28.6%, which is much higher than that of the traditional hydrothermal or solvothermal methods. The XRD pattern (Fig. 1c) depicts two peaks at 21.1° and 42.8°, which correspond to (002) and (100) of graphitic structure. The peak at 21° corresponds to *d* spacing of 0.42 nm, which is larger than the normal *d* spacing of 0.35 nm of the graphitic (002) crystal plane. This implies that the functional groups may exist on the layer surface or edge. Figure 1d shows the typical Raman spectra of the CDs, which exhibit two peaks at 1360 cm^−1^ and 1576 cm^−1^ that contributed from the disordered (D band) and graphite (G band) of carbon materials. The ratio of the *I*_D_/*I*_G_ is 0.98 indicating the CDs possess a degree of graphitization^31^.

**Fig. 2:**
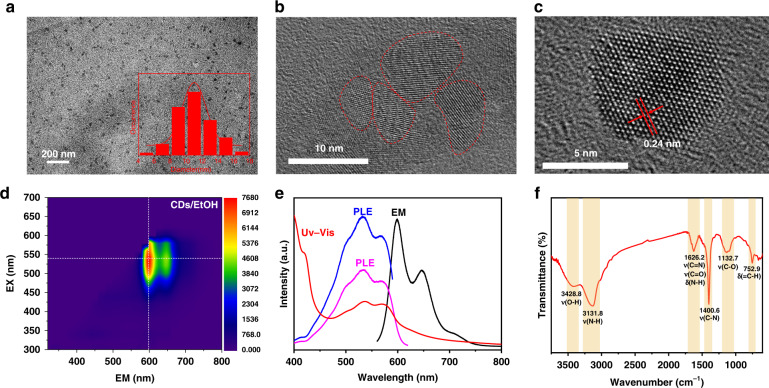
Characterizations of the red emissive CDs. **a** Transmission electron microscopy (TEM) images and the size distribution of CDs. **b** and **c** High-resolution TEM images of CDs. **d** Excitation Emission Matrix (EEM) spectra of CDs dissolved in ethanol. **e** The corresponding UV-vis, PL emission, and PL excitation spectra of CDs dissolved in ethanol. **f** FT-IR spectra of CDs

Transmission electron microscopy (TEM) images (Fig. 2a–c) disclose that obtained CDs are uniform and have a particle size of 10.8 ± 2.3 nm. The high-resolution (HR) TEM images of the CDs disclose the lattice fringe distance was 0.24 nm which is attributed to the *d* spacing of the graphene (100). Figure 2b shows the CDs tend to form an aggregation in the solid state. Figure 2d depicts the excitation-emission matrix (EEM) spectra of CDs in ethanol, which mainly exhibit a 600 nm and 650 nm PL emission center. UV-vis, PL emission and PL excitation (PLE) spectra (Fig. 2e) indicate the emissions at 600 nm and 650 nm originate from the transition of absorption bands at 500, 530, and 570 nm. The absolute photoluminescence quantum yield (QY) is 2.65%. Fourier transform infrared (FTIR) spectra of CDs (Fig. 2e) presents the peaks at 3329, 3132, 1626, 1401, and 1133 cm^−1^ are attributed to the stretching vibrations of O-H, N-H, C = N (or C = O), C-N, and C-O, besides, the peak at 753 cm^−1^ can be assigned to the bending vibration of C-H of the benzene ring. In Fig. S2, the X-ray photoelectron spectroscopy (XPS) analyzed the chemical composition of the CDs. Full-scan XPS spectra (Fig. S2a) reveal the consistency of carbon (C), nitrogen (N), and oxygen (O) elements with an atomic fraction of 85.29%, 9.77%, and 4.84%, respectively. High-resolution XPS spectra of C1s, N1s, and O1s are shown in Fig. S2b, c, d, respectively. The C1s XPS spectra can be deconvoluted into four components: C = C/C-C (284.8 eV), C-N (285.8 eV), C = N (287.2 eV), and C = O (298.0 eV), The N1s XPS spectra can be converted into three types of nitrogen: pyridinic N (C-N = C, 399.1 eV), pyrrolic N (C-NH-C or N-(C)_3_, 400.6 eV), and amine N (N-H, 402.0 eV). Only C = O (532.2 eV) can be observed in O1s XPS spectra^28,29^. These results reveal that dehydration and deamination happen in the formation process of CDs.

### Origin of red emission

Although no solvent was added, the reaction between oPD and CAT happens in homogenous liquid due to the melting points of both oPD and CAT being ~103 °C. To our surprise, when oPD and CAT ethanol solution are mixed at room temperature by the molar ratio of 1:1, the mixed solution gradually turned from colorless to reddish and exhibited red emission with excitation by 532 nm laser (Fig. S3a). EEM spectra, PL emission, and PL excitation spectra show that the emission peaks of the mixed solution are 600 nm and 650 nm, which are very close to the CDs’ emission. Similar optical properties guide us to consider a red emissive molecule formed in the solution, which is the fluorophores of red emissive CDs (Fig. S3d). Consequently, the PL mechanism of this type of CDs should belong to molecule state fluorescent. However, a trace amount of product results in it being hard to determine the molecular structure of the formed molecule.

To investigate the chemical structure of the fluorescent, the oPD and CAT mixture was heated to different temperatures without AlCl_3_·6H_2_O for 6 h to slow down the reaction. The intermediates were investigated by high-performance liquid chromatography-mass spectrometry (HPLC-MS) and Proton Nuclear Magnetic Resonance (^1^H-NMR). Figure S4a shows the HPLC-MS of the product synthesized at 100 °C for 6 h without any further purification (Sample 1). In this mild reaction condition, melt oPD and CAT react to each other. The HPLC graph depicts a strong peak at 0.552 min and two weak peaks at 2.739 min and 3.439 min. The corresponding MS spectra exhibit the peaks of *m/z* at 109, 211, and 285 for the fraction at the retention time of 0.552, 2.739, and 3.439 min, respectively. The estimated molecules are unreacted oPD/CAT, 2,3-DAPN, and DHQP, respectively. ^1^H NMR spectrum of the product (Fig. S4d) displays 4 equal intensity chemical shifts at 6.37, 6.49, 6.60, and 6.72 ppm, and one peak at 8.83 ppm and one at 4.39 ppm. The integration area ratio of these peaks is 1:1:1:1:1:2. The chemical shifts and integration of these peaks completely match the ^1^H NMR peaks of oPD and CAT. Consequently, these peaks are assigned to the H on the unreacted oPD and CAT. Compare to the CAT, the peak at 8.83 ppm (–OH) has changed its shape from the sharp one to a wide weak peak implying that the proton turns more active^32^. Although 2,3-DAPN and DHQP were detected by HPLC-MS, it is hard to be detected by ^1^H NMR due to the amount of these being low.

Figure S4b presents the HPLC graph and the corresponding MS spectra of different fractions for Sample 2 prepared at 200 °C for 12 h. The relative intensity of peaks at both 2.739 min and 3.439 min increases in HPLC spectra (Fig. S4b). In addition, two new peaks appear at the retention time of 4.804 and 5.063 min, the corresponding MS spectra display the *m*/*z* of 181. That retention time and MS peak are the same as those of pure phthalazine (Fig. S4c). ^1^H NMR spectrum of phenazine (Fig. S4d) exhibited two peaks at δ = 7.99 ppm (dd, *J*_1_ = 6.8, *J*_2_ = 3.4 Hz, 4H) and 8.28 ppm (dd, *J*_1_ = 6.7, *J*_2_ = 3.4 Hz, 4H). The same peaks are observed in the green trace (Fig. S4d) implying that phthalazine and reactants are the main product. The other two fractions in HPLC-MS do not detect in the ^1^H NMR spectra of the as-prepared sample. The as-prepared product needs to be further purified to obtain Sample 3.

**Fig. 3:**
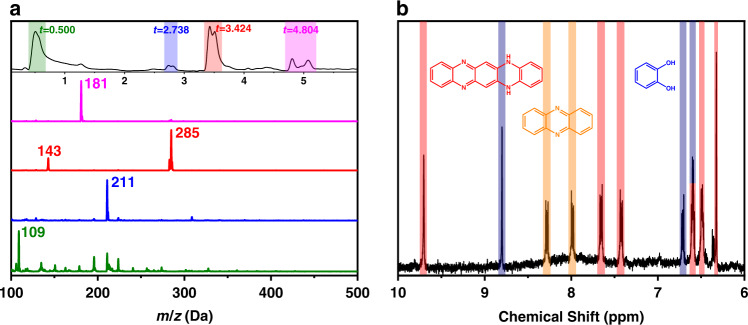
Characterizations of Sample 3. **a** LC-MS of the synthetic Sample 3 (The top illustration is the LC graph; the bottom is the mass spectra corresponding to the peak at 0.500, 2.738, 3.424, and 4.804 min on the chromatogram). **b** ^1^H-NMR of Sample 3

**Fig. 4:**
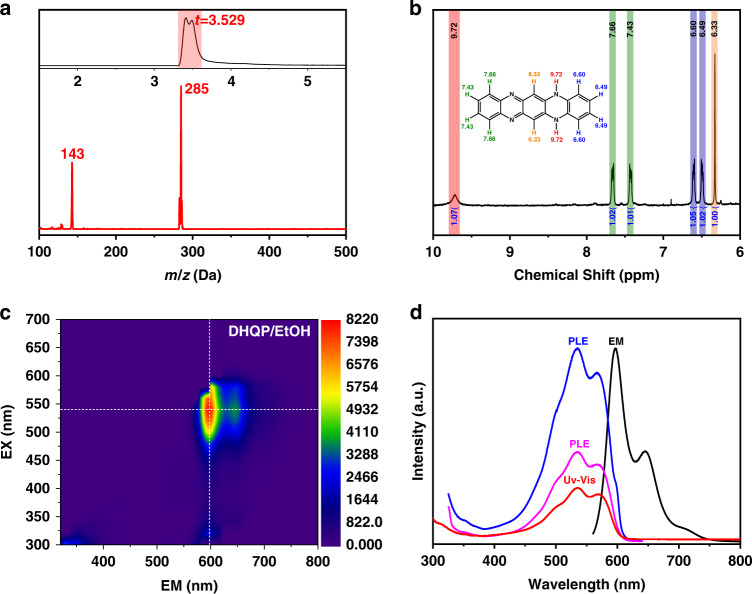
Characterizations of DHQP. **a** LC-MS graph of the synthesized DHQP (The top illustration is the LC graph; the bottom is the MS spectrum corresponding to the retention time of 3.529 min LC graph). **b** ^1^H-NMR of the synthesized DHQP. **c** EEM spectra of DHQP in ethanol. **d** The corresponding UV-vis, PL emission, and PL excitation spectra of DHQP in ethanol

After purification, the HPLC-MS and ^1^H NMR spectra are shown in Fig. 3. The fraction at *t* = 3.424 min significantly increase (Fig. 3a) and the fraction at *t* = 4.804 min decreased due to the impurities being removed. In the corresponding mass spectrometry, The peak at *m*/*z* of 285 is attributed to the DHQP [M + H]^+^. A peak of m/z at 143 emerged, it could correspond to the DHQP molecule carrying 2 charges ([M + 2H]^2+^). The ^1^H NMR spectrum of Sample 3 is presented in Fig. 3b. To determine the attribution of these chemical shifts, DHQP was synthesized with oPD and 2,3-DAPN as a precursor (the detailed synthetic method is given in the ESI). HPLC-MS and ^1^H-NMR were carried out to identify the structure of the synthetic product. The chromatogram spectrum (Fig. 4a) presents a strong signal at 3.529 min, and the corresponding MS spectra emerge two peaks at 285, and 143 Da, which all are characteristic MS peaks of DHQP. The ^1^H NMR spectrum (Fig. 4b) presents the following peaks: 6.32 (s, 2H), 6.49 (dd, *J*_1_ = 5.7, *J*_2_ = 3.4 Hz, 2H), 6.60 (dd, *J*_1_ = 7.3, *J*_2_ = 3.7 Hz, 2H), 7.42 (dd, *J*_1_ = 6.5, *J*_2_ = 3.5 Hz, 2H), 7.66 (dd, *J*_1_ = 6.5, *J*_2_ = 3.5 Hz, 2H), and 9.71 (s, 2H). Compared to ^1^H-NMR spectra of pure DHQP (Fig. 4b), the peaks at 6.32 ppm, 6.45–6.52 ppm, 6.55–6.63 ppm, 7.42 ppm, 7.66 ppm, and 9.70 ppm are contributed to the DHQP in reaction intermediates (Fig. 3b). The chemical shifts at 7.99 ppm and 8.28 ppm are assigned to the phthalazine as well. The peaks at 8.80 ppm and 6.71 ppm are assigned to the H on the –OH and benzene of CAT, the other chemical shift of CAT is at 6.60 ppm, which is coincident with the characteristic chemical peak of DHQP. Based on the above results, the fluorophores of CDs might be the above intermediate molecules such as phenazine, 2,3-DAPN, or DHQP.

To confirm the fluorophores of the CDs, we measured the optical properties of commercial phenazine and 2,3-DAPN (Fig. S5). Phenazine displays a very weak PL emission. The quantum yields are as low as 0.27% in ethanol. The maximum emission of 2,3-DAPN is at 534 nm with an excitation of 420 nm. The PL QY of 2,3-DAPN in ethanol was measured to be 7.05%. These indicate phenazine and 2,3-DAPN are not fluorophores of the CDs. To determine whether or not DHQP is the fluorophore of CDs, The EEM of DHQP is presented in Fig. 4c, which clearly shows two emission centers at 600 nm and 650 nm, which are the same as those of CDs. But the PL QY is about 26.50%, much higher than CDs. UV-vis, PL, and PLE spectra of DHQP are displayed in Fig. 4d, which exhibits two PL emission peaks at 600 and 650 nm, which originate from the transition of absorbance bands at 500, 530, and 570 nm. The PL spectra of DHQP exhibit high similarity to the PL spectra of CDs. We infer that DHQP is the fluorophore of this type of CDs.

**Fig. 5:**
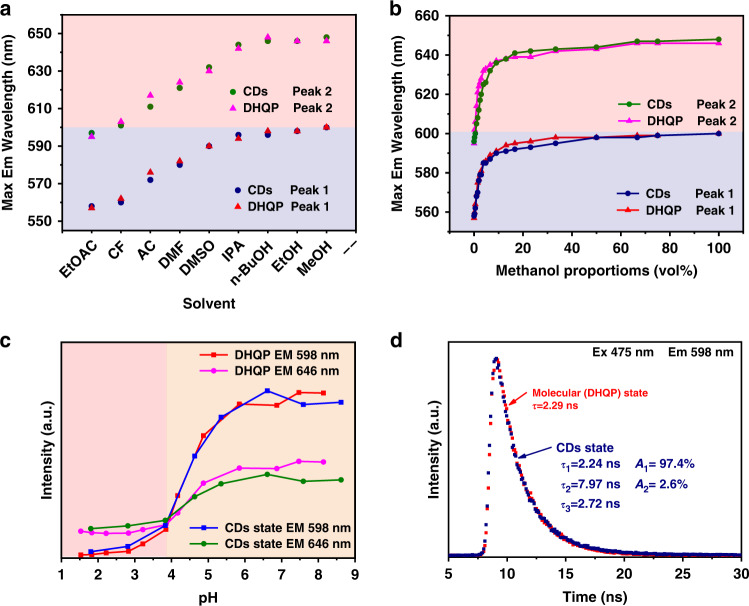
The optical properties of CDs and DHQP. **a** Effect of the solvents on the maximum emission wavelength of the CDs and DHQP under excitation of 540 nm. **b** Changes in the maximal emission wavelength of the CDs and DHQP in methanol/EtOAc with the different methanol proportions. **c** Effect of pH value on the fluorescence intensity at 598 and 646 nm of CDs and DHATP. **d** Time-resolved photoluminescence decay of the emission at 598 nm of CDs and DHQP excited by 475 nm. (EtOAc: Ethyl acetate, CF: Chloroform, AC: Acetone, DMF: N, N-Dimethylformamide, DMSO: Dimethyl sulfoxide, IPA: 2-Propanol, n-BuOH: n-Butanol, EtOH: Ethanol, MeOH: Methanol)

To verify the fluorophore of the CDs is DHQP, we comparative investigate the effect of solvents, pH, and time-resolved PL on the optical properties of CDs and DHQP. Figure S6a, b depict the PL spectra of DHQP and CDs in the common solvent including ethyl acetate (EtOAc), chloroform (CF), acetone (ACE), N, N -dimethylformamide (DMF), dimethyl sulfoxide (DMSO), isopropanol (IPA), n-butyl alcohol (BuOH), ethanol (EtOH), methanol (MeOH). Both CDs and DHQP show a similar shift of two emission peaks (Fig. S5a, b). Figure 5a presents the changing trend of maximal emission with solvent polarity. With decreasing the polarity of the solvent, the maximum emission blue-shift from 600 nm for MeOH with a relative polarity of 0.762 to 558 nm for EtOAc with a relative polarity of 0.228. More important is both CDs and DHQP keep the same pace with changing the relative polarity of the solvent. The phenomenon of the PL peak shifts with changes in solvent polarity may be attributed to a reduced bandgap between the HOMO and LUMO resulting from solvation and the reorientation of polar molecules around the PL molecular group^33,34^. The PL properties of CDs and DHQP was further investigated by tuning solvent polarity by mixing methanol and ethyl acetate. The maximum emission wavelength rapidly shifts toward the long wavelength with the addition of methanol (Fig. 5b). It depicts that a small amount (<2%) of methanol could cause a large redshift from 557 and 595 nm to 577 and 624 nm in DHQP solution, respectively, which is near 50% of the total spectral redshift in pure methanol. And this spectral shift rule is the same as the CDs solution. It is probably induced by hydrogen bonding of hydroxy group on alcohols solvent to the amino groups, rather than general solvent effect^34,35^.

Moreover, the pH effect on the emissions of CDs and DHQP are presented in Fig. S6c, d. With the decrease of pH, the intensity of the PL emission peaks decreases. The emission at 598 nm has a more dramatic descent compared to the peak at 646 nm. When the pH is <4, the emission at 646 nm turns out to be the maximum (Fig. 5c). Overall, the emission of both CDs and DHQP keep the same tune as the pH. Both solvent and pH effects imply that the CDs and DHQP have a very similar optical response to the environment. Time-resolved PL spectra were employed to understand the exciton dynamics of CDs and DHQP. All samples were excited at 475 nm and the emission at 598 nm and 646 nm were probed (Figs. 5c and S7). DHQP molecule exhibits a single-exponential decay implying a single emissive state. The lifetime (*τ*) is 2.29 ns and 2.30 ns for emission at 598 and 646 nm, respectively. The decay traces of CDs are fitted with bi-exponential decays: the short lifetime (*τ*_1_) is 2.24 ns (*A* = 97.4%) and the long lifetime (*τ*_2_) is 7.97 ns (*A* = 2.6%), indicating that an extra process occurs in the red emissive CDs. The short lifetime is very close to the lifetime of DHQP. The long lifetime is attributed to the additional energy transfer from the green and blue units to the neighboring red ones^36^. TEM images disclose that the CDs are composed of a few small conjugated units not a single particle (Fig. 2b). These results indicate both DHQP and CDs have the same origin.

On the other hand, the thermogravimetric analysis (TGA) was carried out to test the thermal stability of DHQP and CDs. As shown in Fig. S8, DHQP exhibits a sharp mass loss within 298 °C. However, the CDs show two significant mass loss peaks at 219 °C and 430 °C. The former may attribute to the removal of the functional group (–OH, –C = O, –NH_2_) on the CDs surface, and the latter may be the further carbonization. Although DHQP and CDs have very similar optical properties, their thermal behavior is quite different, implying no free DHQP is contained in the CDs. To summarize the above results, DHQP and CDs have similar PL properties, which is not originated from the free DHQP in CDs. Consequently, we deduce that DHQP is the fluorophore of this type CDs.

### The formation process of CDs

Based on the above investigation, the three intermediates were produced in the reaction of oPD and CAT at 200 °C. They are 2,3-DAPN, phthalazine, and DHQP based on the HPLC-MS and ^1^H NMR spectra. 2,3-DAPN is the dimer of oPD, and phthalazine is industrially synthesized from oPD and CAT.

**Fig. 6:**
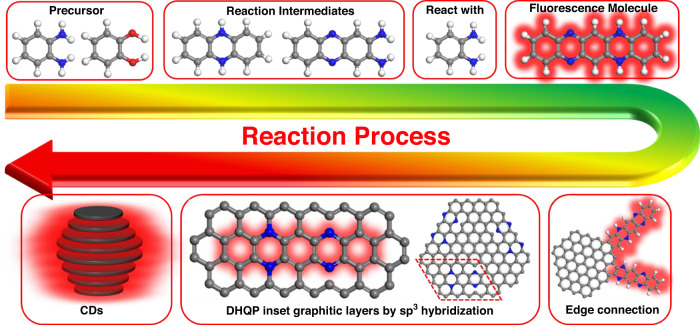
A schematic of the red emission CDs building-up process in the solvent-free method system of oPD and CAT

To fully understand the formation process of DHQP, the oPD and CAT reacted with 2,3-DAPN and/or phthalazine at 100 °C, respectively. The PL spectra of reaction products are shown in Fig. S9, when CAT reacts with 2,3-DAPN and phthalazine, respectively, the PL emission wavelength is 550 and 536 nm, which is not the same as that of DHQP. In contrast, the product from oPD and 2,3-DAPN or phthalazine exhibits 600 and 650 nm emission peaks, which is similar to the DHQP. Accordingly, we speculate on the forming process of the CDs as shown in Fig. 6. First, oPD and CAT react to produce the intermediates (2,3-DAPN and phthalazine). Then, the intermediates further react with oPD to produce DHQP molecule. With the increase of temperature and the extension of reaction time, the molecule grows and carbonizes to form single-layer graphene embedded or connected DHQP molecules to CDs with an sp^3^ bond. The large version of CDs ^1^H-NMR spectra (Fig. S10a) shows that the H of the benzene ring at the DHQP molecular edge significantly disappeared and became weaker. That indicates the benzene ring of the DHQP molecular edge could be incorporated into the structure of the conjugated CDs core (Fig. S10b). Finally, the single-layer graphene stack to zero-dimensional CDs.

### Fluorescent mechanism of CDs

**Fig. 7:**
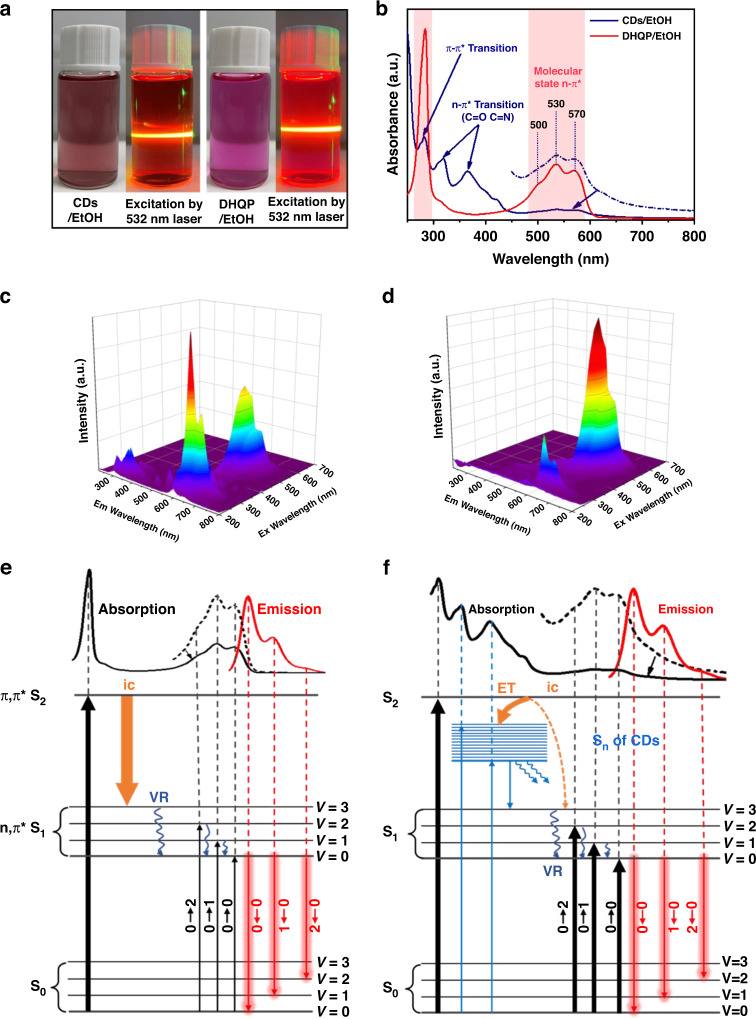
The proposed fluorescent mechanism of DHQP and CDs. **a** Photo of the CDs and DHQP dissolved in EtOH, and the solutions were excited by a 532 nm laser. **b** The UV-vis contrast spectra of the DHQP and CDs. **c** and **d** EEM spectra of DHQP and CDs dissolved in ethanol. **e** and **f** Schematic diagram of the real experimental absorption and emission of DHQP and CDs. (ic: internal conversion, VR: vibration relaxation, ET: electron transfer)

Despite the PL behavior of CDs and DHQP being pretty similar, the UV-vis spectra do not exhibit a high degree of similarity. Firstly, the solutions of CDs in ethanol show dark red and the DHQP solution shows purple (Fig. 7a), implying that they have different UV-vis absorption spectra. Notably, the CDs exhibit unique absorption bands at 318 and 366 nm (Fig. 7b), there are no absorption peaks in the DHQP. Considering the early reports^30,37^, the peaks at 318 and 366 nm could be assigned to the n–π* transition of the C = N and C = O on the CDs surface. In addition, the absorption bands within 480–600 nm and 285 nm are the same as the DHQP, which could be assigned to the n–π* and π–π* transition of the molecular unit, respectively, The EEM spectra of DHQP and CDs are shown in Fig. 7c, d. The excitation wavelength of 285 nm gives more contribution to the emission at 600 nm for the DHQP. On the contrary, the absorption band at 520 nm is the largest contributor to red emission. Figure 7e shows the fluorescent mechanism of DHQP, according to the Frank-Condon principle, the rate of electron transition is much higher than the nucleus motion, the nucleus will move to a new location to adapt to the geometrical configuration of electrons by vibration. Because the vibration is quantized, contributes to generating a lot of vibration energy levels (*V* = 1, *V* = 2, *V* = 3, …) in S_0_ and S_1_ states. Thus, the absorption at 500, 530, and 570 nm and emission bands at 600, 650, and 700 nm are the vibrational overtone bands, which correspond to absorption transition of 0-2, 0-1, 0-0, and emission transition of 0-0, 0-1, 0-2, respectively. The 0-1 transition is the most probable Frank-Condon transition^30,38,39^. And the π–π* transition at 285 nm corresponds to the energy level transition between the S_0_ and S_2_, the excited electron decay to the lowest vibration energy levels of S_1_ through internal conversion (IC) and vibration relaxation, then return to S_o_ through radiative emission transition of 0-0, 0-1, 0-2 (Fig. 7e). That is the reason why the excitation of 285 nm gives more contribution to the red emission.

In the case of CDs, DHQP was connected or embedded with carbon core by sp^3^ matrix, and the fluorophore (DHQP) on the CDs is ultimately dominant in photoluminescent behaviors. The PL mechanism of 600, 650, and 700 nm emission excited by the 480–600 nm band is the same as those in DHQP. Intriguingly, the absorption peak at 285 nm has less contribution to the red emission, the probable reason is the excited electron may transfer to the energy level of CDs (S_n_) which is mostly a non-radiation transition. The new excited energy levels (S_n_) locate between S_1_ and S_2_ states related to the absorption band at 318 nm and 366 nm. The electron of S_2_ can transfer to the S_n_ of the CDs, which competes with internal conversion between S_2_ and S_1_ (Fig. 7f). This causes the PL intensity excited by the 285 nm band to be decreased.

## Discussion

In summary, we synthesized the red emission CDs in high yield through the solvent-free method, and analyzed the origin of red emission and forming process of CDs. We separated the intermediates during the synthesis of the CDs by HPLC-MS. HPLC-MS and ^1^H NMR spectra determine the intermediates containing the 2,3-DAPN, phthalazine, and DHQP. It reveals that the PL mechanism of this type of CDs should belong to molecule state fluorescent by comparing the PL properties of intermediates and the CDs. The PL emission and absorption are almost the same as the CDs in the visible region, the PL lifetime, PL change behavior in different solvents, and pH of DHQP are the same as the CDs, and the fluorophore of the CDs is DHQP, which incorporate into graphene or connect with graphene edge by sp^3^ hybridization. During the PL emission of CDs, the electron of molecule state S_2_ transfer to the S_n_ of CDs, resulting in the 285 nm exciting PL intensity at 480–600 nm band decreased. Our findings can potentially help understand the PL mechanism of the CDs that synthesized used oPD as a precursor and inspire a novel synthetic design to obtain red emission with tailored properties.

## Materials and methods

### Materials

o-Phenylenediamine, catechol, and aluminum chloride hexahydrate were purchased from Aladdin. phenazine and 2,3-diaminophenazine were purchased from Energy Chemical and Heowns, respectively. All chemicals were used without further purification.

### Synthesis of red emission CDs

The red emission CDs were prepared through the solvent-free method (Fig. 1a) the processes are as follows: 0.54 g (5 mmol) oPD, 0.55 g (5 mmol) CAT, and 0.08 g (0.33 mmol) AlCl_3_·6H_2_O were mixed and ground uniformly in an agate mortar for 10 min. Then the mixture was transferred to a 30 mL Teflon-lined autoclave and heated for 12 h at 200 °C. Subsequently, the autoclave was taken out and cooled to room temperature naturally. The obtained carbonized powders were dissolved in an ethanol solution and filtered through a 0.22 μm polyethersulfone membrane to remove large particles. And finally, the filtered solution was dialyzed in a dialysis bag with a cut-off of 3500 Da against DI water for 7 d, and the DI water was changed every 12 h.

### Synthesis of samples 1, 2, and 3

Sample 1: 0.54 g oPD and 0.54 g CAT were mixed and ground uniformly in an agate mortar, and then the mixture was transferred to a 30 mL Teflon-lined autoclave for heating for 6 h at 100 °C. Sample 2: 0.54 g oPD and 0.54 g CAT were mixed and ground uniformly in an agate mortar, and then the mixture was transferred to a 30 mL Teflon-lined autoclave and heated for 12 h at 200 °C. Sample 3: Sample 2 was dissolved in 200 mL ethanol, the solution was centrifuged at 10,000 rpm for 10 mins, and the solid was selected to vacuum drying at 40 °C.

### HPLC MS sample preparation

The Sample was dissolved in DMSO to form the 1 mg/mL solution. Then, the solution was diluted 100 times with water, acetonitrile and trifluoroacetic acid (95/5/0.1) to be measured. The model of HPLC-MS is Agilent 1290 Infinity/6460 LC/QQQ MS. C18 column (2.1 × 50 mm, 1.7 μm) was used to separate with the mobile phase A of 0.1% formic acid-water and mobile phase B of 0.1% formic acid-acetonitrile. The separation products were ionized in the electrospray ionization (ESI) and operated in positive mode.

## Supplementary information

supplementary file
