# Supplementary material for: Formation and fluorescent mechanism of red emissive carbon dots from o-phenylenediamine and catechol system
Source: Light Sci Appl. 2022 Oct 13;11:298. doi: 10.1038/s41377-022-00984-5 (PMC9561683; doi:10.1038/s41377-022-00984-5)
Supplement: Supplementary file 1 — supplementary file [file 41377_2022_984_MOESM1_ESM.docx]

Supplementary materials for

Formation and fluorescent mechanism of red emissive carbon dots from o-phenylenediamine and catechol system

Pengfei Li, Shanshan Xue, Lu Sun, Xupeng Zong, Li An, Dan Qu, Xiayan Wang and Zaicheng Sun*

Center of Excellence for Environmental Safety and Biological Effects, Beijing Key Laboratory for Green Catalysis and Separation, Department of Chemistry and Chemical Engineering, Beijing University of Technology, Beijing 100124, China.

Email: sunzc@bjut.edu.cn

**Experimental section**

**Synthesis of DHQP**

2.3 g of 2,3-DAPN (10.9 mmol) and 1.9 g of oPD (17.6 mmol) were added into a 100 mL radius flask, 20 mL of benzyl alcohol as a solvent to reflux at 210℃ for 20 h. After the reaction is finished, the solid was collected by vacuum filtration. It was refluxed in a 10% H_2_SO_4_ solution. After that, the solid was collected by vacuum filtration, and it was neutralized and washed with a mixed solution of ethanol and ammonia (V_EtOH_: V_ammonia_ =39:l) to obtain a purple-black solid. Finally, the solid was dry in a vacuum oven at 60°C overnight.

**Characterization**

The UV-Vis absorption spectra were recorded using the Shimadzu UV-2600 spectrophotometer. Steady-state fluorescence (PL) spectra were collected on HITACHI F-7000 fluorescence spectrophotometer from the excitation wavelength range of 300 to 700 nm. The crystal structure of the CDs was investigated using X-ray diffraction (XRD; Bruker D8 Advance X-ray diffractometer) with Cu Kα radiation (λ=0.15406 nm) as the incident beam at 40 kV and 40mA. Raman spectra were recorded on Lab Ram HR Raman microscope. The CDs morphology was observed by transmission electron microscopy (TEM, JEOL JEM-2100F equipped) with a LaB6 source operating at 200 kV. High-resolution TEM images were obtained with the same TEM equipment. Fourier transform infrared (FTIR) spectra were measured by Bruker Vertex 70 spectrometer. The surface composition and elemental chemical state of CDs were examined by X-ray photoelectron spectroscopy (XPS; Thermo Scientific K-Alpha) equipped with an Al Ka monochromator X-ray source. Thermal properties were measured by Thermogravimetric (TGA, labs Evo) under a nitrogen atmosphere from room temperature to 1000°C with a heating rate of 10°C min^-1^. Fluorescence lifetimes were characterized using Edinburgh FLS1000 time-corrected single-photon counter system with 475 nm excitation wavelength. ^1^H NMR spectra were recorded in DMSO-d6 on an ASCEndTM-400 MHz spectrometer (Bruker AVANCE HD III) using tetramethylsilane (TMS) as an internal standard.


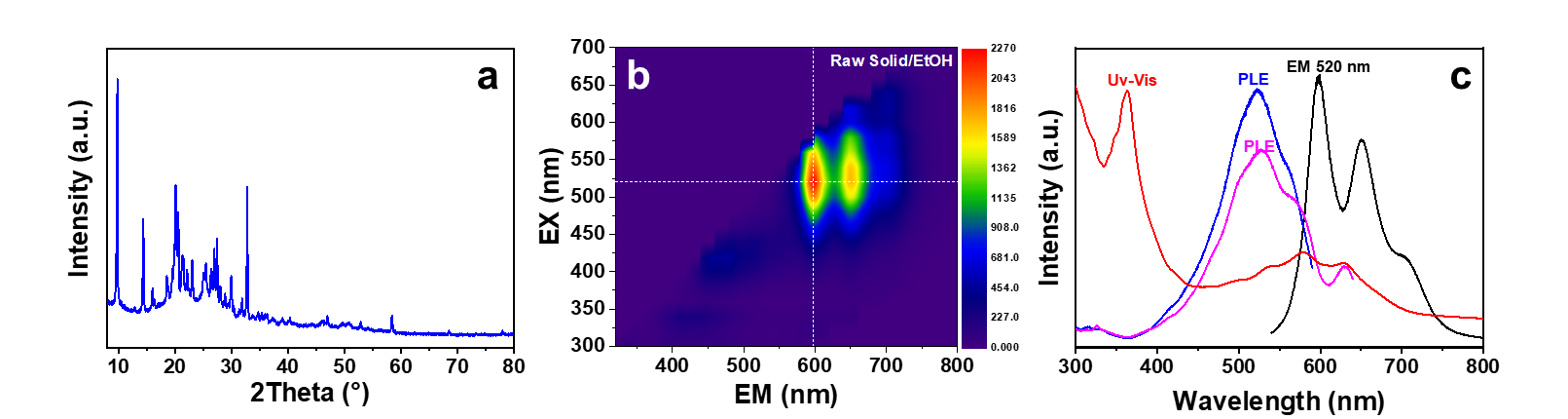


Fig. S1 **a** XRD pattern of the raw solid. **b** Excitation Emission Matrix (EEM) spectra raw solid dissolved in ethanol. **c** The corresponding UV-vis, PL emission, and PL excitation spectra of raw solid dissolved in ethanol.


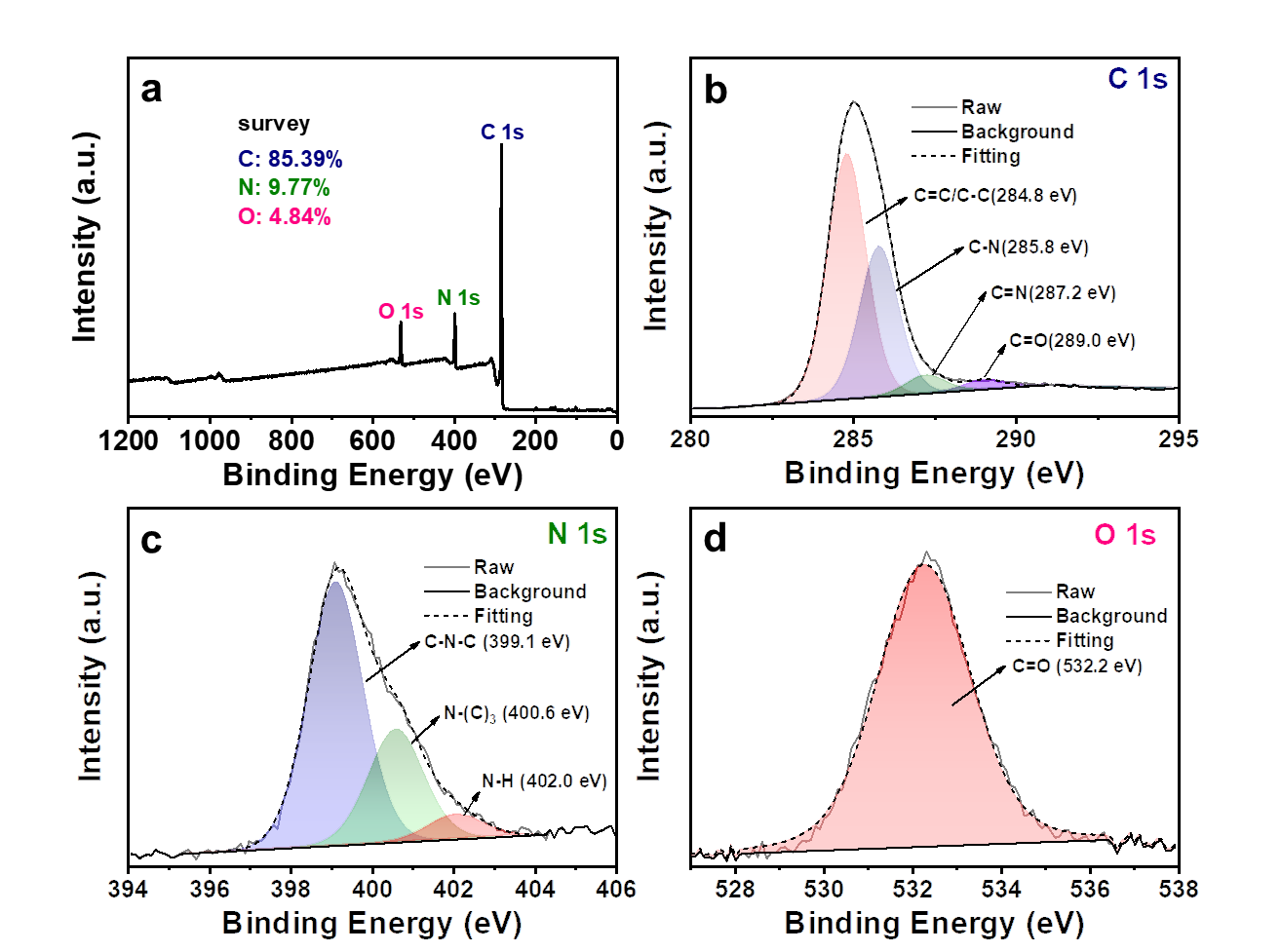


**Fig. S2** **a** Full-scale XPS survey spectrum of CDs. **b-d** The high-resolution C 1s, N 1s, and O 1s XPS spectra of CDs.


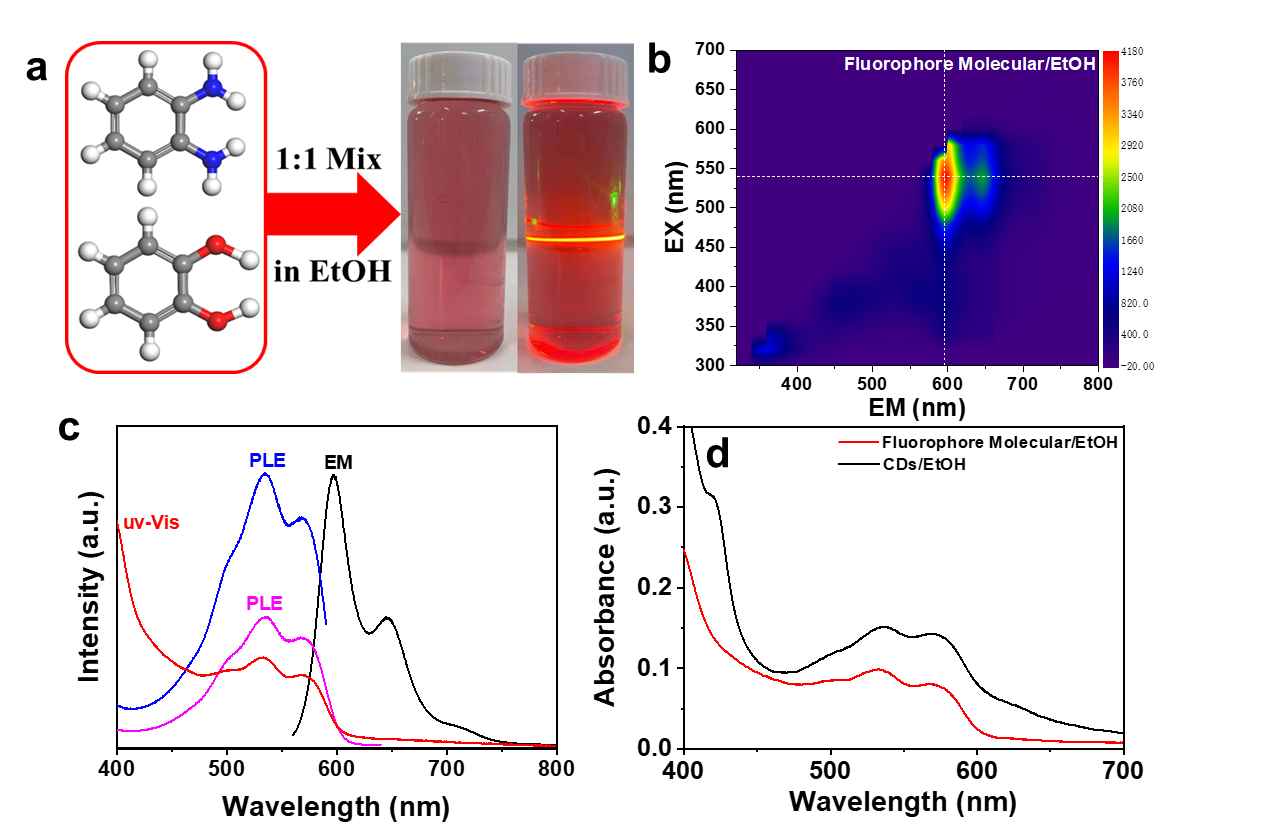


**Fig. S3** **a** Photo of the OPDA and CAT mixed dissolve by 1:1 in EtOH. **b** EEM spectra of the mixed solution of OPDA and CAT. **c** The corresponding UV-vis, PL emission, and PL excitation spectra mixed solution of OPDA and CAT. **d** UV-vis spectra of mixed solution and A-CDs.


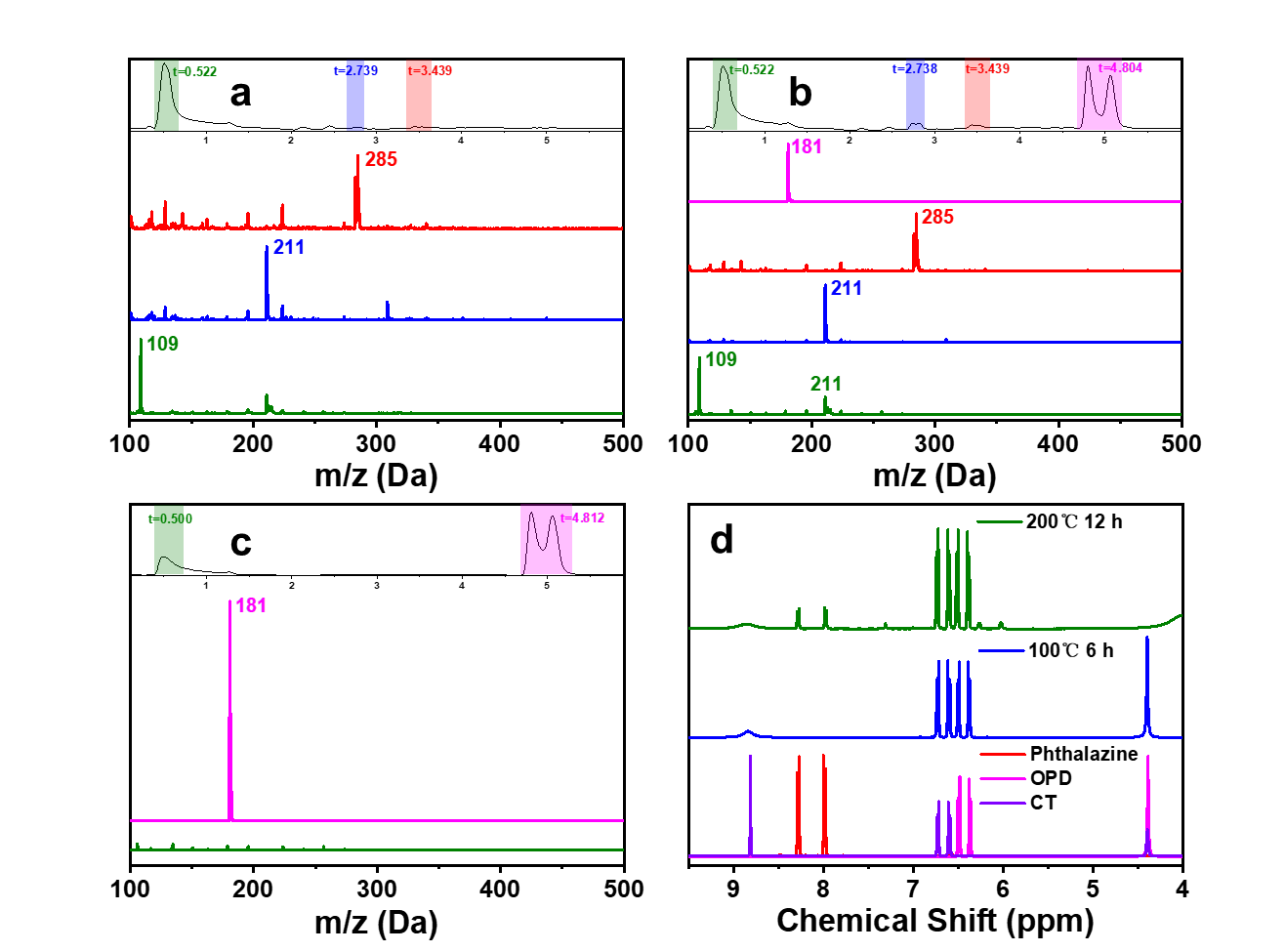


**Fig. S4** **a-c** LC-MS of the reaction product from OPD and CAT at 100℃ for 6 h (Sample 1, **a**), 200℃ for 12 h (Sample 2, **b**), pure phthalazine, **c**. the top illustration is liquid chromatogram, the bottom is the mass spectra corresponding to the chromatogram. **d** 1H-NMR of the reaction product in different reaction conditions.


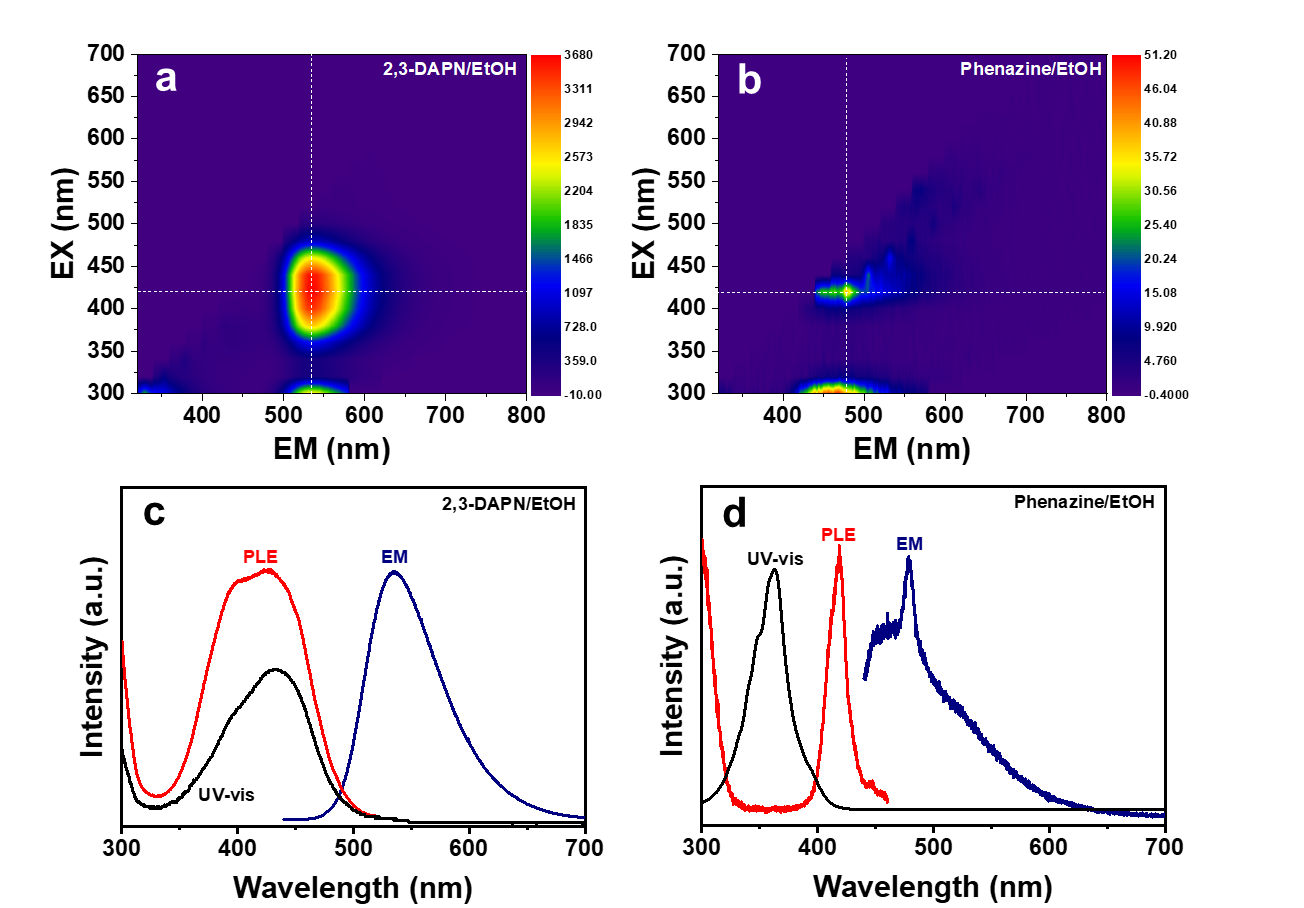


**Fig. S5** Optical properties of phenazine and 2,3-DAPN. **a, b** EEM spectra of 2,3-DAPN and phenazine dissolved in ethanol. **c, d** The corresponding UV-vis, PL emission, and PL excitation spectra of 2,3-DAPN and phenazine dissolved in ethanol.


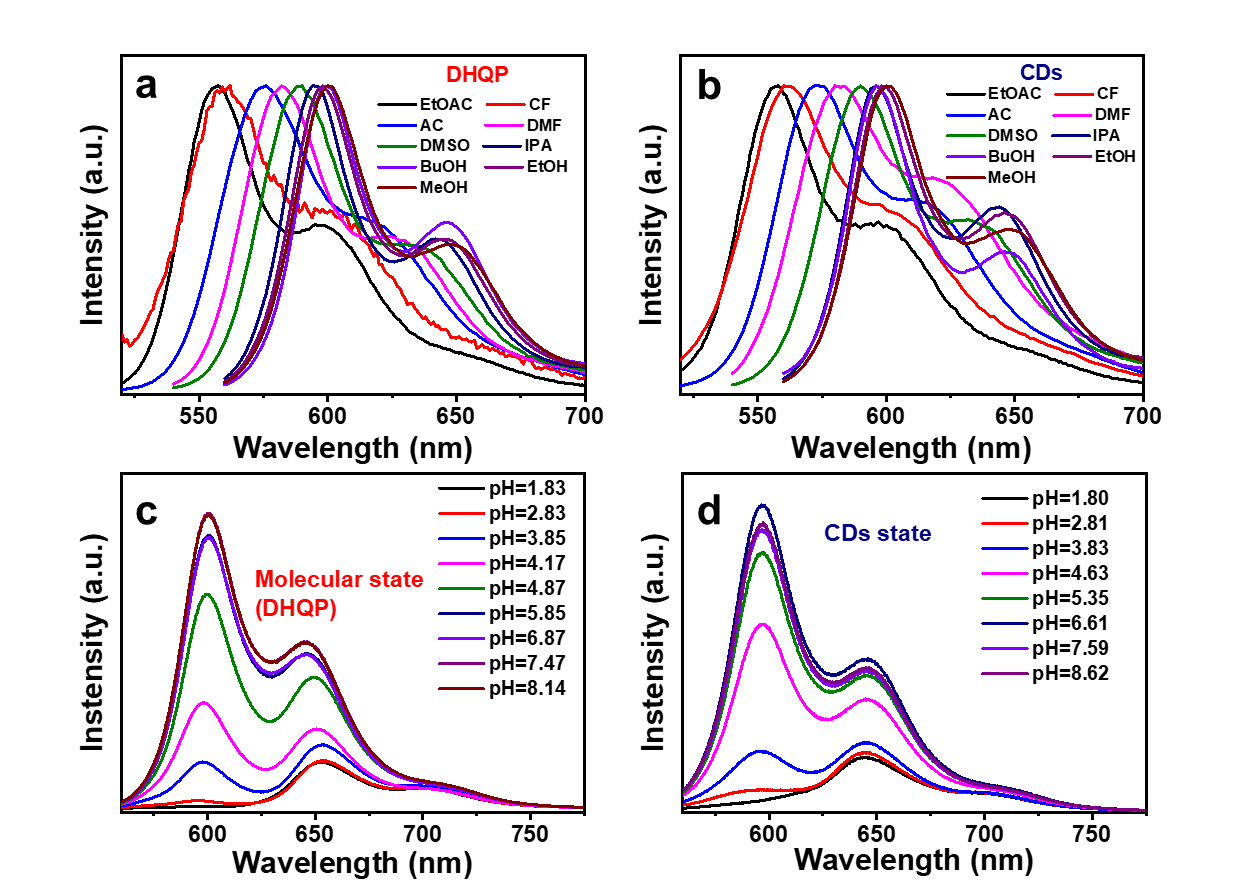


**Fig. S6** **a, b** Normalized fluorescence spectra of DHQP **a** and CDs **b** under 540 nm excitation in different solvents. **c, d** Typical emission spectra of DHQP **c** and CDs **d** at different pH.


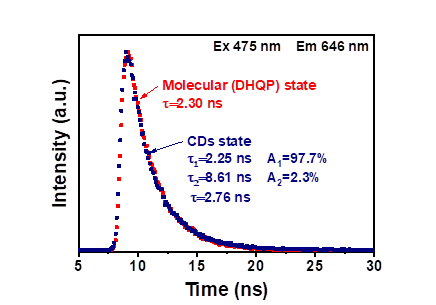


**Fig. S7** Fluorescence decay spectra excited by 475 nm of CDs and DHTAP at 598 nm.


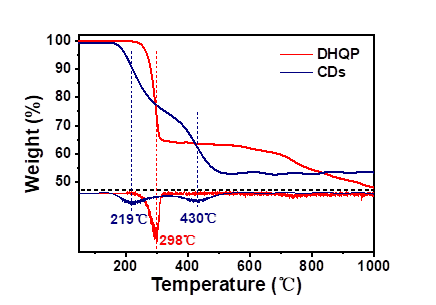


**Fig. S8** TGA spectrum of CDs and DHTAP (the illustration below is the weight loss peaks of CDs and DHTAP through the derivation of the TGA data.


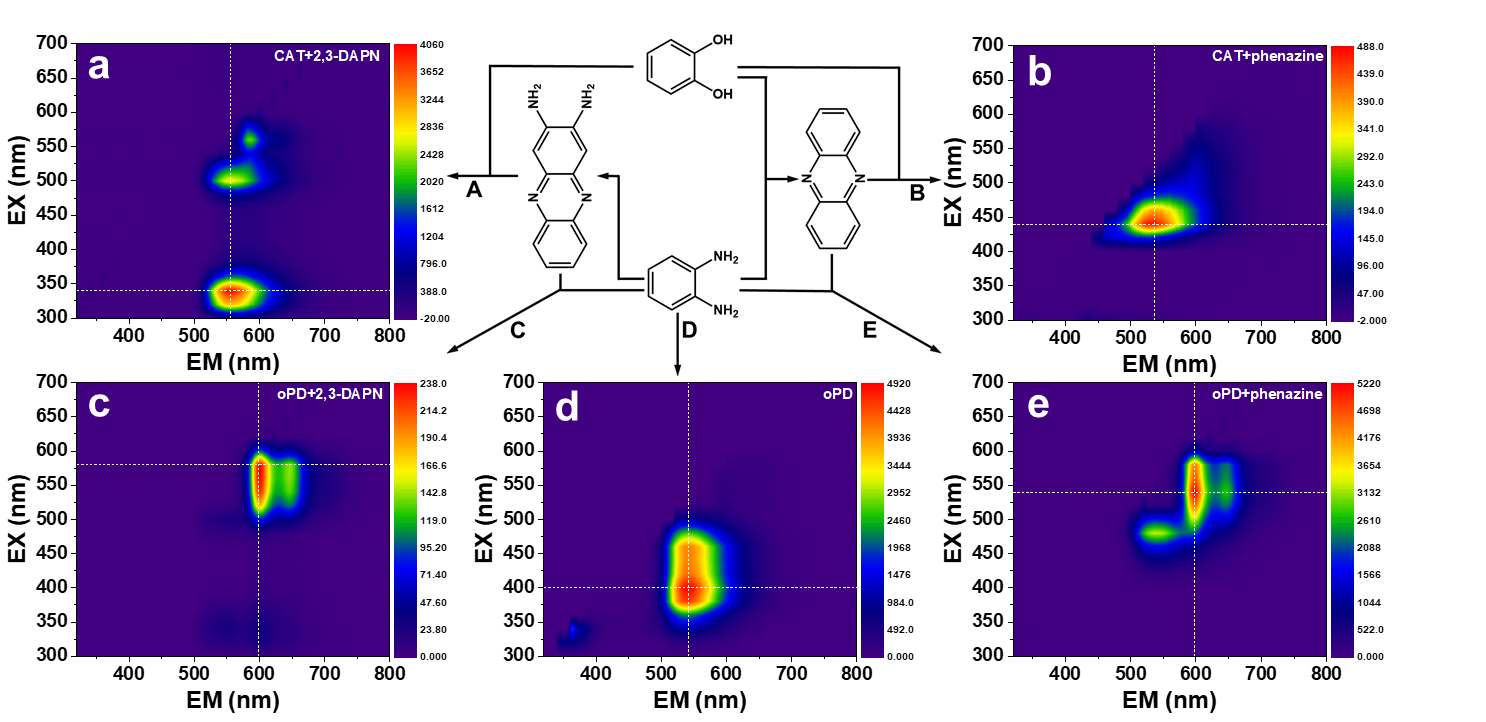


**Fig. S9 The EEM spectra of the reaction product from precursors and intermediates.** **a** CAT and 2,3-DAPN. **b** CAT and phenazine. **c** oPD and 2,3-DAPN. **d** only oPD. **e** oPD and phenazine.


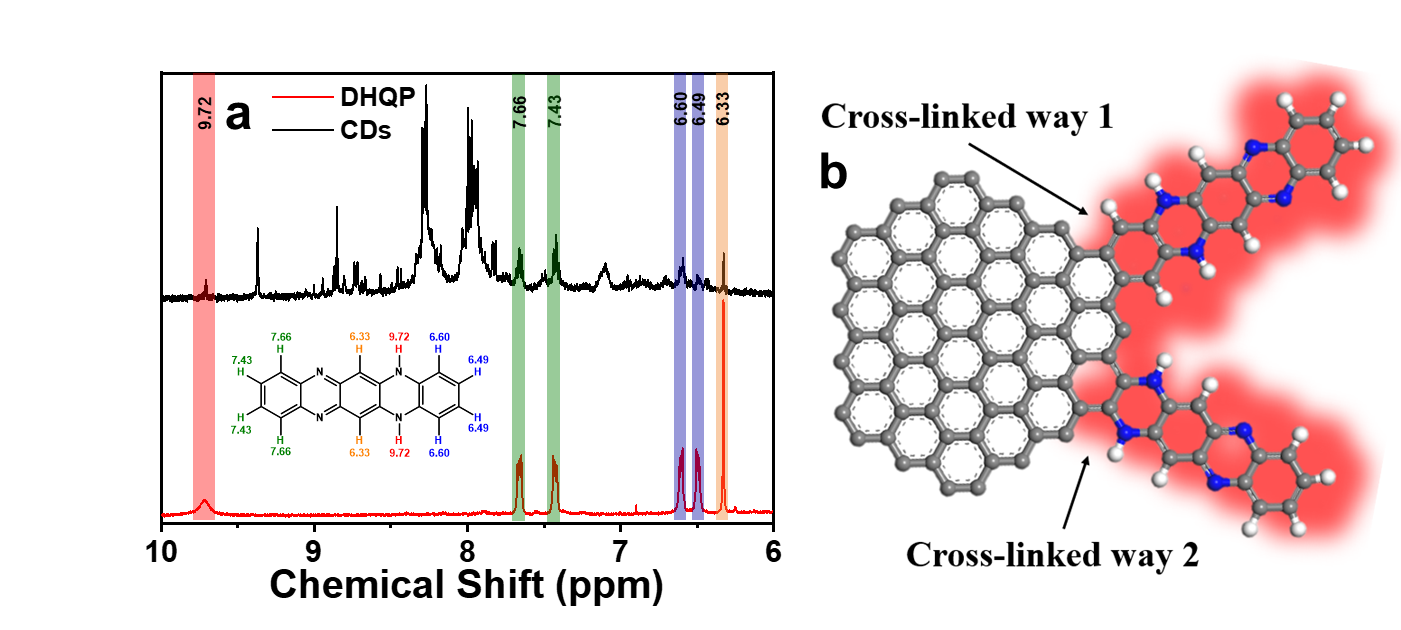


**Fig. S10 a** The large version of CDs 1H-NMR spectra at 6 ppm-10 ppm. **b** The schematic of the DHQP molecule cross-linked with the CDs.

The range between 6 ppm and 10 ppm of CDs' 1H NMR spectra was magnified 100 times to analyze the H on the conjugated part of CDs (Figure R1 d). That present many irregular peaks that may attribute from the H of the core edge and molecular group. The chemical shift peaks at 6.33, 6.49, 6.60, 7.43, 7.66, 9.72 ppm of CDs were certainly compared with the 1H NMR spectra of DHQP. The four peaks at 6.49, 6.60, 7.43, and 7.66 ppm attributed to the C-H on edge benzene rings have almost equal intensity in DHQP (Figure S10 a). The chemical shifts at 6.49 and 6.60 ppm (blue frames) exhibit relatively weaker intensity than the peaks at 7.43 and 7.66 ppm (green frames). Even the intensity of peak at 6.49 ppm is lower than that of peak at 6.60 ppm. Those imply that benzene ring (green edge) lost their H. Consequently, we infer that the DHQP might chemically link with carbon dots as shown in Figure S10 b.
